# Editorial

**Published:** 1913-06

**Authors:** 


### EDITORIAL

The Business Office of the *Journal-Record* is 23 West Alabama Street.

The Editorial Office is 1014-15 Atlanta National Bank Building.

Address all Business Communications to *Journal-Record of Medicine,* 23 West Alabama Street.

Make remittances payable to *The Journal-Record of Medicine.*

On matters pertaining to the Editorial and Original Communications, address Edgar G. Ballenger, M. D., Atlanta, Ga.

Reprints of Original Articles will be furnished at cost price. Requests foi the same should always be made in THE MANUSCRIPT.

We will present, postpaid, on request to each contributor of an original article, twenty (20) marked copies of *The Journal-Record of Medicine* containing such article.

### THE ALKALINE TREATMENT OF ACUTE NEPHRITIS AND POST-OPERATIVE NEPHRITIS.

Elsewhere in this issue we publish in full a valuable paper by Dr. Martin H. Fischer on "The Treatment of Nephritis and Allied Conditions" by carbonate of soda. His theory as to the etiology of these affections is based upon the idea that an excessive acidity is an active cause of acute and chronic parenchymatous nephritis. Whether the physician subscribes to all of Fischer's views or not, a test of this remedy in appropriate cases will convince him of its value. In the light of our present knowledge it would seem reprehensible to allow patients to die of acute nephritis, eclampsia, and such conditions without giving this remedy a chance, according to the exigencies of the case. The alkaline treatment during and after scarlet fever might prevent the nephritis which so frequently follows.

The writer has been using this treatment for about eighteen months and has had quite satisfactory results from it. Albumin and tube casts have cleared from the urine at times with astonishing rapidity, remain diminished or absent during the treatment and may or may not return when it is discontinued. Patients have thus been enabled to secure life insurance who had been declined on account of the presence of casts and albumin. In one patient the albumin, when precipitated by the Esbach method, equaled one-half the volume of urine; fifteen to twenty tube casts could be found in the average low-powerfield. Within four days after the institution of this treatment the albumin had diminished to about one-twentieth the original amount and the tube casts to two or three to the field. The patient had first found the albuminuria when declined by an insurance company; he had been in a hospital on a rigid diet and rest with little improvement three months previously.

Another history of interest is that of a patient, fifty-seven years of age, from whom we removed the prostate gland. His condition was satisfactory until four days after the operation when he developed a sore throat and sore mouth which prevented his drinking liquids with comfort. Hiccough, evidencies, uremia and final suppression of the urine developed a few days later in spite of the usual measures to combat such conditions. There were the usual symptoms, as hiccough, drowsiness, a pinched, drawn facial expression and no urine. He was given 1200 c. c. of the alkaline solution at five P. M. Within four hours the hiccough stopped. During the next nine hours we collected and measured fifteen ounces of urine. Four more intravenous injections were given during the next seven days. After this the renal function continued as good as it was before the operation. This patient's kidneys were seriously impaired and when first seen the phthalein test required thirty minutes to appear in the urine. We are convinced that without the Fischer treatment this patient would have died.

E. G. Ballenger, M. D.

---

### SHALL WE PIT SALVARSAN AGAINST MERCURY OR BOTH AGAINST SYPHILIS?

The important question for one to decide in the treatment of syphilis should not be "mercury versus salvarsan" but rather how may both be employed to secure a clinical cure and lasting negative Wassermann and luetin tests with the minimum harm to the patient.

Among the most harmful effects of the mercurial treatment first and foremost are the dangers of uncured syphilis and parasyphilis. Whether these are avoidable or unavoidable evils, when considered from the oretical standpoint need not enter the discussion, for history is replete with evidence that in actual every day practice they must be considered ever present dangers. Can we hope soon to develope more skill and judgement on the part of the physician or more patience and persistence on the part of the patient? When boasting of the wonderful potency of mercury should we not remember that the frequency of tabes, paresis, aneurysm, many cardiac lesions, optic atrophy, much of deafness and many minor complaints are real dangers which in the past have been inseperable from the mercurial treatment? Are they not grewsome milestones marking it's inadequacy for four-hundred years? Is not this inadequacy it's greatest danger? What of the few hundred deaths that have followed the few million administrations of salvarsan compared with the hosts that have died of uncured syphilis? The blood tests of more than half of the patients who have received what we formerly considered a fairly good course of treatment with mercury (2 or 3 years) are still postive, some after ten, twenty and thirty years. Is not the prolonged tedious course required also one of the real and constantly present dangers of the mercurial treatment? Compare the health of the patient who has received at suitable intervals six or eight intravenous injections of medium size doses of salvarsan, which have cured the clinical evidenses of the disease and rendered the serologic tests negative, with ehalth of the patient who has taken sufficient mercury and iodides to secure the same result. Such a comparison will show, except in rare instances, that the health, weight and feeling of well being of the patient treated with salvarsan are far more satisfactory than of the one treated with mercury. We have under observation now, hundreds of patients whose condition is quite as good as it was before syphilsi was contracted. From a study of syphilis in years gone by, when we depended largely upon mercury and the iodides, must we not admit that uncured early or late syphilitic and parasyphilitic affections are among the greatest of the dangers from treatment with these remedies? How can we avoid such an admission? All medical works upon this disease as well as the records of hospitals and insane asylums prove it.

Are we to be free from uncured syphilis and parasyphilis after the salvarsan treatment? Certainly not if the course be inadequate and not checked by serologic tests. On the other hand though we have every reason to assume that those who have remained clinically well with persistently negative blood tests for the past few years will continue well. We can at least say that formerly we have never been able to secure anything like such uniform results, negative Wassermann reactions, and clinical cures free from relapses with the mercurial treatment as we have with salvarsan.

Evident advantages of salvarsan are the prompt healing of lesions from which syphilis might be spread to others, the rapid cessation of all active luetic manifestions, the more lasting negative Wassermann reactions when the doses are appropriately repeated, the much less disagreeable treatment, the shorter time required to effect the cure or at least render all tests negative, and finally the good health of these patients when compared with the various complaints of the patients who have received prolonged treatment with mercury. The new infections with syphilis which we have observed in five patients treated with salvarsan indicates we think that these patients were completely cured or such infections would not have occured.

A few theoretical pessimists predict that we are to have many atrophic nerve lesions and dire consequences in years to come as a result. We venture to hope they will not occur. We at least have in toxicology no instance where a chemical could remain quiescent for years and then suddenly or slowly produce damage of such a peculiar nature. As nearly the total quantity of salvarsan is eliminated within one or two weeks after it's intravenous injection we can hardly take seriously any such prediction as regards future harm from it.

We might with equal reason fear salivation from five grains of calomel one or ten years after its administration and elimination because this amount of mercury in a more potent form would be sure to salivate or poison the patient. Such hair splitting with the eminent and real dangers of uncured syphilis before us merits little consideration. Again we assert that salvarsan and mercury should not be pitted against each other, but both should be used carefully and scientifically to fight syphilis and parasyphilis.

### OFFICERS OF THE MEDICAL ASSOCIATION OF GEORGIA.

At the sixty-fourth annual meeting of the Medical Association of Georgia held in Savannah, April 16-18, the following officers were elected: president, Dr. Ralston Lattimore, Savannah; vice-presidents, Drs. J. D. Chason, Bainbridge, and S. R. Roberts, Atlanta; secretary-treasurer, Dr. W. C. Lyle, Augusta (re-elected); delegate to the American Medical Association, Dr. M. A. Clark, Macon; alternate, Dr. C. T. Nolan, Marietta, and councilors, Dr. W. L. Champion, Atlanta, fifth district; Dr. J. R. B. Branch, Macon, sixth district; Dr. H. Clay Willis, Rome, seventh district, and Dr. Edward Gallard Adams, Greensboro, eighth district. Dr. William B. Hardman, Commerce, was re-elected chairman, and Dr. J. Lawton Hiers, Savannah, secretary of the council. Atlanta was selected as the next place of meeting.

